# A novel IFN-CSM-CoCoSo approach for multiple-attribute group decision-making with intuitionistic fuzzy sets: An application in assessing corporate social responsibility performance

**DOI:** 10.1016/j.heliyon.2024.e29207

**Published:** 2024-04-06

**Authors:** Jing Nie

**Affiliations:** School of Accounting, Jilin Business and Technology College, Changchun, 130507, Jilin, China

**Keywords:** Multiple-attribute group decision-making, Intuitionistic fuzzy sets, CoCoSo technique, CRITIC technique, Performance evaluation

## Abstract

With the rapid growth of the economy, enterprises have encountered a series of problems while pursuing economic benefits, such as food safety and environmental pollution issues, resource shortages and energy consumption issues, which affect the sustainable development of enterprises. Establishing a corporate performance evaluation system from the perspective of social responsibility, based on stakeholder theory and the importance of overall goals reflected in the weight of social responsibility indicators, is a very effective measure to achieve corporate social responsibility (CSR) goals through CSR motivation and stakeholders. The performance evaluation of CSR from the perspective of environmental accounting is a MAGDM. Recently, the CoCoSo technique and cosine similarity measure (CSM) technique was utilized to conduct the MAGDM. The intuitionistic fuzzy sets (IFSs) are utilized as a technique for conducting uncertain information during the performance evaluation of CSR from the perspective of environmental accounting. In this study, the intuitionistic fuzzy CoCoSo based on the CSM (IFN-CSM-CoCoSo) technique is built for MAGDM with IFSs. Finally, a numerical example for performance evaluation of CSR from the perspective of environmental accounting is conducted to verify the IFN-CSM-CoCoSo technique.

## 1 Introduction

At the beginning of the 20th century, the concept of CSR first produced in the United States. In 1924, Irvine Shelton first advocated the concept of "CSR" from an academic perspective, which means that enterprises should not only obtain profits and bear corresponding laws and regulations for shareholders and personnel, but also take responsibility for customers, organizations, and the environment. The social responsibility requirement of enterprises is different from the traditional theory that regards profit as the sole goal [[1], [2], [3], [4]]. It emphasizes the attention to human value in the production process and the contribution made to the customers and society. As a result, people began to pay attention to the social responsibility of companies in various fields [[5], [6], [7]]. In terms of CSR performance, according to earlier research in the West, it has been established that the true purpose of performance evaluation is to inspect, supervise, and conduct enterprises [8,9]. The research on performance evaluation in China can be roughly divided into three periods-the formation period, the improvement period, and the innovation and development period of performance evaluation. In the view of this article, CSR performance is the study of relationship between corporate management level and corporate performance, which uses corporate indicators to reflect the degree of contribution to society [[8], [9], [10], [11]]. At present, some scholars [[12], [13], [14], [15], [16]] have studied the performance evaluation of coal enterprises on social responsibility from multiple perspectives. Shen, Govindan and Shankar [17] started from the perspective of social responsibility in the balanced scorecard and utilized the Analytic Hierarchy Process to make decisions on production safety, social environment, and sustainable development. From the Attig, El Ghoul, Guedhami and Suh [18] researched on the social performance of commercial banks, social performance includes a series of investment behaviors and internal behaviors of the company, mainly focusing on the development of employee welfare and fair treatment of employees. Similar to social interest issues, such as community relations, charitable projects, etc., are considered a multifaceted concept. Ye and Li [19] conducted the empirical analysis on relationship between economic performance and environmental performance of highly polluting enterprises. The results indicate that firstly, economic performance has positive impact on environmental performance; Secondly, there is a positive correlation between environmental performance and economic performance [19]. In summary, most literature on CSR performance focuses on stakeholder perspectives, reflecting CSR performance issues through financial indicators and production and operation conditions [[20], [21], [22], [23]]. There is a lack of exploration of CSR performance from the perspective of environmental accounting, and there is a lack of multi-dimensional empirical research. In quantitative analysis, different literatures often uses factor analysis to explore the reasons, with relatively limited techniques [[24], [25], [26]]. Therefore, this article combines the characteristics of coal enterprises and, from the perspective of environmental responsibility, combines financial indicators with environmental indicators. Compared to previous studies, it has a more comprehensive performance dimension and differs from traditional perspectives in selecting specific financial indicators. It increases the evaluation of environmental indicators on enterprise performance, making the results more objective, accurate, and comprehensive [27,28].

MAGDM is an important component of the decision-making field, mainly solving limited alternative decision-making problems containing multiple attributes [[29], [30], [31], [32], [33], [34], [35]]. Its characteristic is to evaluate a limited number of alternative solutions through a certain method based on the evaluation information provided by the DMs, and then obtain a ranking [[36], [37], [38], [39], [40], [41], [42], [43], [44]]. In the MAGDM process, the evaluation value of the solution is a given value [45,46]. However, the knowledge and experience of decision-makers, strategic vision, personal preferences and values, attitude towards risk, and complexity of the environment can all affect the optimal ranking of solutions [[47], [48], [49], [50], [51], [52], [53]].

The performance evaluation of CSR from the perspective of environmental accounting is MAGDM. Recently, the CoCoSo [[54], [55], [56], [57], [58], [59], [60]] and CSM [[61], [62], [63], [64], [65], [66], [67], [68], [69]] was utilized to conduct MAGDM. The IFSs [[70], [71], [72], [73]] are an efficient tool for conducting uncertain information during the performance evaluation of CSR from the perspective of environmental accounting. Until now, no or few techniques were studied on CRITIC [[74], [75], [76], [77], [78], [79], [80]]and CoCoSo in light with CSM under IFSs. Therefore, in this study, the IFN-CSM-CoCoSo is conducted for MAGDM with IFSs. Finally, numerical study for performance evaluation of CSR from the perspective of environmental accounting is validated the proposed technique. The main research motivation of this study is outlined: (1) the CoCoSo technique was extended to IFSs in light with CSM; (2) the CRITIC technique is utilized to derive weight with CSM under IFSs. (3) the IFN-CSM-CoCoSo technique is conducted the MAGDM with IFSs; (4) numerical example for performance evaluation of CSR from the perspective of environmental accounting and comparative analysis is validated the IFN-CSM-CoCoSo.

The framework of this study is conducted. In Sect. 2, the IFSs is conducted. In Sect. 3, IFN-CSM-CoCoSo is conducted under IFSs with CRITIC. Sect. 4 conducted the numerical example for performance evaluation of CSR from the perspective of environmental accounting and comparative analysis. The conclusion is done in Sect. 5.

## 2 Preliminaries

Atanassov [81] done the IFSs.

**Definition 1** [81]. The IFSs is conducted:

(1) $$L=\left\{\left\langle \theta ,u_{L}\left(\theta \right),v_{L}\left(\theta \right)\right\rangle |\theta \in \Theta \right\}$$

where $\mu_{L}\left(\theta \right)\in \left[0,1\right]$ is membership and $\nu_{L}\left(\theta \right)\in \left[0,1\right]$ is non-membership: $0\leq \mu_{L}\left(\theta \right)+\nu_{L}\left(\theta \right)\leq 1$, ∀θ∈Θ. Then, lθ=(lu,lv) is conducted as the IFN.

**Definition 2** [82,83]. Let $l\theta_{1}=\left(lu_{1},lv_{1}\right)$ and $l\theta_{2}=\left(lu_{2},lv_{2}\right)$ be IFNs, the SF (score function) and AF(accuracy function) is conducted:

(2) $$LSF\left(l\theta_{1}\right)=lu_{1}-lv_{1},LSF\left(l\theta_{2}\right)=lu_{2}-lv_{2}$$

(3) $$LAF\left(l\theta_{1}\right)=lu_{1}+lv_{1},LAF\left(l\theta_{2}\right)=lu_{2}+lv_{2}$$

For two IFNs $l\theta_{1}=\left(lu_{1},lv_{1}\right)$ and $l\theta_{2}=\left(lu_{2},lv_{2}\right)$, then [84]

$$\begin{array}{c} \left(1\right)\;if\;LSF\left(l\theta_{1}\right)<LSF\left(l\theta_{2}\right),l\theta_{1}<l\theta_{2};\\ \left(2\right)\;if\;LSF\left(l\theta_{1}\right)=LSF\left(l\theta_{2}\right),LAF\left(l\theta_{1}\right)<LAF\left(l\theta_{2}\right),l\theta_{1}<l\theta_{2};\\ \left(3\right)\;if\;LSF\left(l\theta_{1}\right)=LSF\left(l\theta_{2}\right),LAF\left(l\theta_{1}\right)=LAF\left(l\theta_{2}\right),l\theta_{1}=l\theta_{2}. \end{array}$$

**Definition 3** [85]. Let $l\theta_{1}=\left(lu_{1},lv_{1}\right)$ and $l\theta_{2}=\left(lu_{2},lv_{2}\right)$ be IFNs, the IFN distanced measure (IFNDM) is supplied:

(4) $$IFNDM\left(l\theta_{1},l\theta_{2}\right)=\frac{1}{2}\left[\begin{array}{c} \left|\frac{2\left(lu_{1}l\pi_{2}-lu_{2}l\pi_{1}-4\left(lu_{1}-lu_{2}\right)\right)}{4-l\pi_{1}l\pi_{2}}\right|\\ +\left|\frac{4\left(lv_{1}-lv_{2}\right)+2\left(lv_{1}l\pi_{2}-lv_{2}l\pi_{1}+2\left(l\pi_{1}-l\pi_{2}\right)\right)}{4-l\pi_{1}l\pi_{2}}\right| \end{array}\right]$$

where $l\pi_{1}=1-lu_{1}-lv_{1},l\pi_{2}=1-lu_{2}-lv_{2}$.

**Definition 4** [84]. Let $l\theta_{1}=\left(lu_{1},lv_{1}\right)$ and $l\theta_{2}=\left(lu_{2},lv_{2}\right)$ be IFNs, several operations are conducted:

(5) $$l\theta_{1}⊕l\theta_{2}=\left(lu_{1}+lu_{2}-lu_{1}lu_{2},lv_{1}lv_{2}\right)$$

(6) $$l\theta_{1}⊗l\theta_{2}=\left(lu_{1}lu_{2},lv_{1}+lv_{2}-lv_{1}lv_{2}\right)$$

(7) $$\zeta l\theta_{1}=\left(1-{\left(1-lu_{1}\right)}^{\zeta },{\left(lv_{1}\right)}^{\zeta }\right),\zeta >0$$

(8) $${\left(l\theta_{1}\right)}^{\zeta }=\left({\left(lu_{1}\right)}^{\zeta },1-{\left(1-lv_{1}\right)}^{\zeta }\right),\zeta >0$$

The IFWA and IFWG technique is done.

**Definition 5** [86]. Let $l\theta_{j}=\left(lu_{j},lv_{j}\right)$ be IFNs, the IFWA technique is done:

(9) $$IFWA_{lw}\left(l\theta_{1},l\theta_{2},\ldots ,l\theta_{n}\right)=\overset{n}{\underset{j=1}{⊕}}\left(lw_{j}l\theta_{j}\right)=\left(1-\prod_{j=1}^{n}{\left(1-lu_{j}\right)}^{lw_{j}},\prod_{j=1}^{n}{\left(lv_{j}\right)}^{lw_{j}}\right)$$

where $lw={\left(lw_{1},lw_{2},...,lw_{n}\right)}^{T}$ be weight of $l\theta_{j}$, $lw_{j}>0,\sum_{j=1}^{n}lw_{j}=1\text{.}$.

**Definition 6** [87]. Let $l\theta_{j}=\left(lu_{j},lv_{j}\right)$ be IFNs, the IFWG technique is done:

(10) $$IFWG_{lw}\left(l\theta_{1},l\theta_{2},\ldots ,l\theta_{n}\right)=\overset{n}{\underset{j=1}{⊗}}{\left(l\theta_{j}\right)}^{lw_{j}}=\left(\prod_{j=1}^{n}{\left(lu_{j}\right)}^{lw_{j}},1-{\prod_{j=1}^{n}\left(1-lv_{j}\right)}^{lw_{j}}\right)$$

where $lw={\left(lw_{1},lw_{2},...,lw_{n}\right)}^{T}$ be weight of $l\theta_{j}$, $lw_{j}>0,\sum_{j=1}^{n}lw_{j}=1\text{.}$.

## 3 IFN-CSM-CoCoSo technique for MAGDM

Then, IFN-CSM-CoCoSo is conducted for MAGDM. Let $LA=\left\{LA_{1},LA_{2},\cdots ,LA_{m}\right\}$ be alternatives, and attributes $\mathrm{LG}=\left\{LG_{1},LG_{2},\cdots ,LG_{n}\right\}$ with weight $lw={\left(lw_{1},lw_{2},...,lw_{n}\right)}^{T}$, where $lw_{j}\in \left[0,1\right]$, $\sum_{j=1}^{n}lw_{j}=1$ and experts $LE=\left\{LE_{1},LE_{2},\cdots ,LE_{q}\right\}$ with weight $l\omega ={\left(l\omega_{1},l\omega_{2},...,l\omega_{n}\right)}^{T}$.

**Fig. 1:**
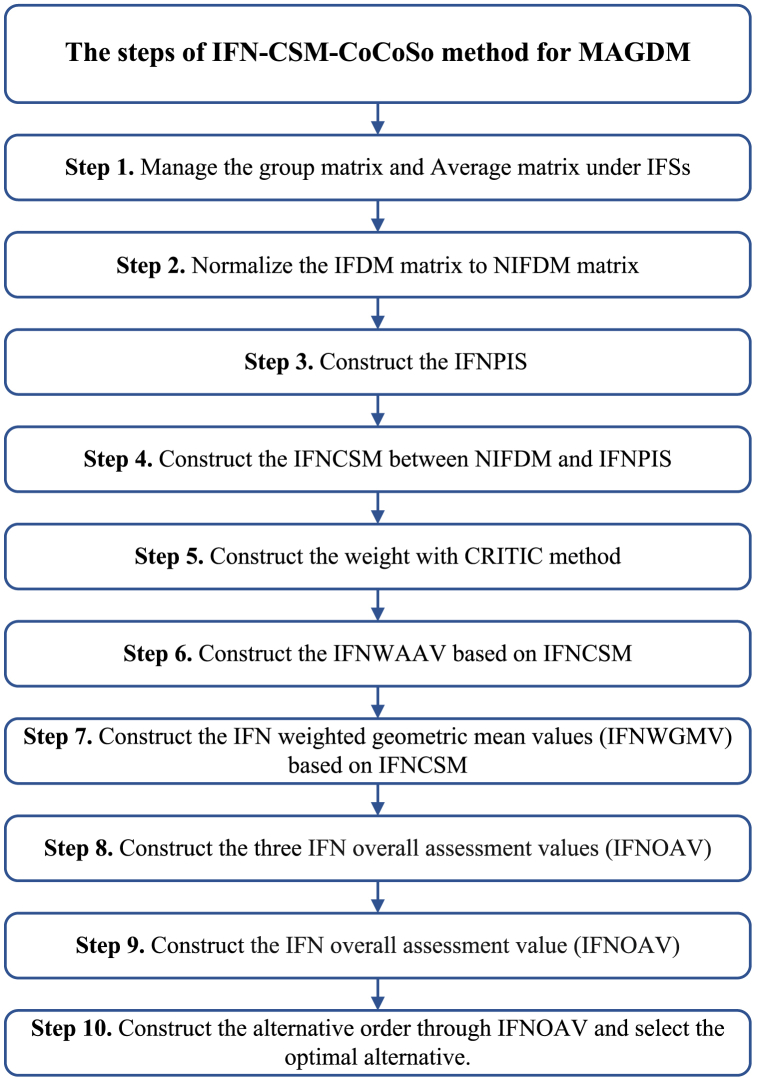
The steps of IFN-CSM-CoCoSo technique for MAGDM.

Then, IFN-CSM-CoCoSo is conducted for coping with the MAGDM (See Fig. 1).

Step 1 Construct the group matrix $IFDM^{t}={\left[IFDM_{ij}^{t}\right]}_{m\times n}={\left(lu_{ij}^{\left(t\right)}，lv_{ij}^{\left(t\right)}\right)}_{m\times n}$ and average matrix $IFDM={\left[IFDM_{ij}\right]}_{m\times n}$:

(11) $$\begin{array}{c} \begin{array}{cccc} \;LG_{1} & \;LG_{2} & \;\ldots & \;LG_{n} \end{array}\\ IFDM^{t}={\left[IFDM_{ij}^{t}\right]}_{m\times n}=\begin{array}{c} LA_{1}\\ LA_{2}\\ \vdots \\ LA_{m} \end{array}\left[\begin{array}{cccc} IFDM_{11}^{t} & IFDM_{12}^{t} & \ldots & IFDM_{1n}^{t}\\ IFDM_{21}^{t} & IFDM_{22}^{t} & \ldots & IFDM_{2n}^{t}\\ \vdots & \vdots & \vdots & \vdots \\ IFDM_{m1}^{t} & IFDM_{m2}^{t} & \ldots & IFDM_{mn}^{t} \end{array}\right] \end{array}$$

(12) $$\begin{array}{c} \begin{array}{cccc} \;LG_{1} & \;LG_{2} & \;\ldots & \;LG_{n} \end{array}\\ IFDM={\left[IFDM_{ij}\right]}_{m\times n}=\begin{array}{c} LA_{1}\\ LA_{2}\\ \vdots \\ LA_{m} \end{array}\left[\begin{array}{cccc} IFDM_{11} & IFDM_{12} & \ldots & IFDM_{1n}\\ IFDM_{21} & IFDM_{22} & \ldots & IFDM_{2n}\\ \vdots & \vdots & \vdots & \vdots \\ IFDM_{m1} & IFDM_{m2} & \ldots & IFDM_{mn} \end{array}\right] \end{array}$$

In light with IFWA technique, the $IFDM={\left[IFDM_{ij}\right]}_{m\times n}={\left(lu_{ij},lv_{ij}\right)}_{m\times n}$ is:

(13) $$IFDM_{ij}=l\omega_{1}IFDM_{ij}^{1}⊕l\omega_{2}IFDM_{ij}^{2}⊕\cdots ⊕lS\omega_{q}IFDM_{ij}^{q}=\left(1-\prod_{t=1}^{q}{\left(1-lu_{ij}^{\left(t\right)}\right)}^{l\omega_{t}},\prod_{t=1}^{q}{\left(lv_{ij}^{\left(t\right)}\right)}^{l\omega_{t}}\right)$$

Step 2 Normalize the $IFDM={\left[IFDM_{ij}\right]}_{m\times n}={\left(lu_{ij},lv_{ij}\right)}_{m\times n}$ into $NIFDM={\left[NIFDM_{ij}\right]}_{m\times n}={\left(nlu_{ij},nlv_{ij}\right)}_{m\times n}$.

Aimed at benefit attributes:

(14) $$NIFDM_{ij}=\left(nlu_{ij},nlv_{ij}\right)=\left(lu_{ij},lv_{ij}\right)$$

Aimed at cost attributes:

(15) $$NIFDM_{ij}=\left(nlu_{ij},nlv_{ij}\right)=\left(lv_{ij},lu_{ij}\right)$$

Step 3 Construct the IFN positive ideal solution (IFNPIS):

(16) $$IFNPIS_{j}=\left(nlu_{j},nlv_{j}\right)$$

(17) $$LSF\left(IFNPIS_{j}\right)=\max_{i}LSF\left(nlu_{ij},nlv_{ij}\right)$$

Step 4 Obtain the IFNCSM between $NIFDM_{ij}$ and $IFNPIS_{j}$.

(18) $$IFNCSM\left(NIFDM_{ij},IFNPIS_{j}\right)=\cos\left[\frac{\pi }{4}\left(\left|nlu_{ij}-nlu_{j}\right|+\left|nlv_{ij}-nlv_{j}\right|+\left|nl\pi_{ij}-nl\pi_{j}\right|\right)\right]$$

(19) $$nl\pi_{ij}=1-nlu_{ij}-nlv_{ij},nl\pi_{j}=1-nlu_{j}-nlv_{j}$$

Step 5 Construct the weight in line with existing CRITIC.

The CRITIC [74] is done to get the objective weight. The calculation steps are conducted through the CRITIC.

(1) Obtain the IFN correlation coefficient (IFNCC):

(20) $$IFNCSM\left(NIFDM_{j},IFNPIS_{j}\right)=\frac{1}{m}\sum_{i=1}^{m}IFNCSM\left(NIFDM_{ij},IFNPIS_{j}\right)$$

(21) $$IFNCC_{jk}=\frac{\left(\begin{array}{c} \sum_{i=1}^{m}\left(IFNCSM\left(NIFDM_{ij},IFNPIS_{j}\right)-IFNCSM\left(NIFDM_{j},IFNPIS_{j}\right)\right)\cdot \\ \left(IFNCSM\left(NIFDM_{ik},IFNPIS_{k}\right)-IFNCSM\left(NIFDM_{k},IFNPIS_{k}\right)\right) \end{array}\right)}{\sqrt{\begin{array}{c} \sum_{i=1}^{m}{\left(IFNCSM\left(NIFDM_{ij},IFNPIS_{j}\right)-IFNCSM\left(NIFDM_{j},IFNPIS_{j}\right)\right)}^{2}\\ {\sum_{i=1}^{m}\left(IFNCSM\left({NIFDM}_{ik},IFNPIS_{k}\right)-IFNCSM\left({NIFDM}_{k},IFNPIS_{k}\right)\right)}^{2} \end{array}}}$$

(2) Construct the IFN standard deviation (IFNSD) and the index (IFNSDC):

(22) $$IFNSD_{j}=\sqrt{\frac{1}{m-1}\sum_{i=1}^{m}{\left(\begin{array}{c} IFNCSM\left(NIFDM_{ij},IFNPIS_{j}\right)\\ -IFNCSM\left(NIFDM_{j},IFNPIS_{j}\right) \end{array}\right)}^{2}}$$

(23) $$IFNSDC_{j}=IFNSD_{j}\sum_{k=1}^{n}\left(1-IFNCC_{jk}\right),j=1,\ldots ,n\text{.}$$

(3) Get the objective weight:

(24) $$lw_{j}=\frac{IFNSDC_{j}}{\sum_{j=1}^{n}IFNSDC_{j}},j=1,\ldots ,n\text{.}$$

Step 6 Construct the IFN weighted arithmetic average values (IFNWAAV) based on IFNCSM.

(25) $$IFNWAAV_{i}=\sum_{j=1}^{n}lw_{j}\times IFNCSM\left(NIFDM_{ij},IFNPIS_{j}\right)=\sum_{j=1}^{n}lw_{j}\times \cos\left[\frac{\pi }{4}\left(\left(\left|nlu_{ij}-nlu_{j}\right|+\left|nlv_{ij}-nlv_{j}\right|+\left|nl\pi_{ij}-nl\pi_{j}\right|\right)\right)\right]$$

Step 7 Construct the IFN weighted geometric mean values (IFNWGMV) based on IFNCSM.

(26) $$IFNWGMV_{i}=\sum_{j=1}^{n}{\left(IFNCSM\left(NIFDM_{ij},IFNPIS_{j}\right)\right)}^{lw_{j}}=\sum_{j=1}^{n}{\left(\cos\left[\frac{\pi }{4}\left(\left(\left|nlu_{ij}-nlu_{j}\right|+\left|nlv_{ij}-nlv_{j}\right|+\left|nl\pi_{ij}-nl\pi_{j}\right|\right)\right)\right]\right)}^{lw_{j}}$$

Step 8 Construct the three IFN overall assessment values (IFNOAV):

(27) $$IFNOAV_{i}\left(a\right)=\frac{IFNWAAV_{i}+IFNWGMV_{i}}{\sum_{i=1}^{m}\left(IFNWAAV_{i}+IFNWGMV_{i}\right)}$$

(28) $$IFNOAV_{i}\left(b\right)=\frac{IFNWAAV_{i}}{\min_{i}IFNWAAV_{i}}+\frac{IFNWGMV_{i}}{\min_{i}IFNWGMV_{i}}$$

(29) $$IFNOAV_{i}\left(c\right)=\frac{\lambda IFNWAAV_{i}+\left(1-\lambda \right)IFNWGMV_{i}}{\lambda \max_{i}IFNWAAV_{i}+\left(1-\lambda \right)\max_{i}IFNWGMV_{i}},0\leq \lambda \leq 1$$

Step 9 Construct the IFN overall assessment value (IFNOAV).

(30) $$IFNOAV_{i}=\sqrt[{3}]{IFNOAV_{i}\left(a\right)\times IFNOAV_{i}\left(b\right)\times IFNOAV_{i}\left(c\right)}+\frac{IFNOAV_{i}\left(a\right)+IFNOAV_{i}\left(b\right)+IFNOAV_{i}\left(c\right)}{3}$$

Step 10 Construct the alternative order through $IFNOAV_{i}$, and the higher $IFNOAV_{i}$, the optimal alternative $LA_{i}$ is.

## 4 Numerical example and comparative analysis

### 4.1 Numerical example

With the development of society, the issue of CSR has become increasingly prominent, attracting widespread attention from the society. Since the 21st century, academic research on CSR has gradually increased and deepened. Currently, the decision relationship between CSR and corporate performance has been constantly debated. From a single perspective of enterprise development, under traditional economic theory, some scholars believe that if the improvement of profits is not the sole goal, it will prevent the owners and shareholders of the enterprise from seeking maximum enterprise value, thus leading to a negative correlation between CSR and corporate performance. Some scholars believe that while enterprises undertake social responsibility, the waste of resources can also affect their competitiveness in the fiercely competitive economic market, resulting in a lack of competitive advantage and inability to establish themselves in the market. However, with rapid development of economy and pursuit of economic benefits by enterprises, failure to balance social and ecological development will result in the loss of sustainable development ability due to other objective factors. Based on stakeholder theory, more and more scholars have found that the beneficiaries of healthy and stable development of enterprises are not only business owners and investors, but also consumers of enterprise products or services. With the rapid development of the economy, problems such as resource shortage and environmental pollution are becoming increasingly prominent, resulting in insufficient resources for enterprise economic development, and the inability to achieve sustainable development in social conditions and natural environment. The proposal of signal transmission theory has formed a dynamic balance between the fulfillment of CSR and the interests of various stakeholders, which can promote information equivalence between corporate development and social demands, and is conducive to the construction of a comprehensive corporate performance evaluation system. Social audiences and corporate investors can make comprehensive and objective evaluations of enterprises through a comprehensive corporate performance evaluation system, identify trustworthy enterprises, and provide richer resource advantages and competitive advantages for the development of enterprises. In addition, by building a corporate performance system, enterprises can better supervise their level of social responsibility, accumulate a large amount of intangible moral capital for enterprise development, and when facing development risks, investors can objectively view the problems of enterprise development from a long-term perspective, helping enterprises improve risk management. The performance evaluation of CSR from the perspective of environmental accounting is a MAGDM. There are five high-tech enterprises $LA_{i}\left(i=1,2,3,4,5\right)$ to select with different attributes [19]: LG_1_ is innovation performance; LG_2_ is management costs; LG_3_ is social performance; LG_4_ is ecological performance. Five high-tech enterprises $LA_{i}\left(i=1,2,3,4,5\right)$ are evaluated based on linguistic scales (Table 1 [88]) in light with different attributes through three experts $LE_{t}\left(t=1,2,3\right)$ with weight vector (0.34,0.40,0.26).

**Table 1:** Linguistic scales and IFNs [88].

Linguistic scales	IFNs
Exceedingly Terrible-LET	(0.1000,0.8000)
Very Terrible-LVT	(0.2000,0.7000)
Terrible-LT	(0.3000,0.6000)
Medium-LM	(0.5000,0.5000)
Well-LW	(0.6500,0.3000)
Very Well-LVW	(0.7500,0.2000)
Exceedingly Well-LEW	(1.0000,0.0000)

The IFN-CSM-CoCoSo technique is conducted performance evaluation of CSR from the perspective of environmental accounting.

Step 1 **.** Manage the $IFDM^{t}={\left[IFDM_{ij}^{t}\right]}_{5\times 4}\left(t=1,2,3\right)$ (See Table 2, Table 3, Table 4).

**Table 2:** Linguistic scales by $LE_{1}$.

	LG_1_	LG_2_	LG_3_	LG_4_
LA_1_	LVW	LVT	LW	LM
LA_2_	LVW	LM	LT	LW
LA_3_	LVT	LM	LM	LM
LA_4_	LM	LVT	LM	LW
LA_5_	LT	LVW	LW	LVT

**Table 3:** Linguistic scales by $LE_{2}$.

	LG_1_	LG_2_	LG_3_	LG_4_
LA_1_	LM	LVW	LT	LVW
LA_2_	LVT	LM	LVW	LVT
LA_3_	LVT	LVW	LW	LT
LA_4_	LVW	LW	LVW	LT
LA_5_	LVW	LT	LVT	LM

**Table 4:** Linguistic scales by $LE_{3}$.

	LG_1_	LG_2_	LG_3_	LG_4_
LA_1_	LT	LW	LM	LW
LA_2_	LVW	LW	LVT	LVT
LA_3_	LM	LW	LT	LVW
LA_4_	LM	LT	LVW	LW
LA_5_	LVT	LM	LW	LM

In light with IFWA technique, the $IFDM={\left[IFDM_{ij}\right]}_{5\times 4}$ is conducted (See Table 5).

**Table 5:** The $IFDM={\left[IFDM_{ij}\right]}_{5\times 4}$.

	LG_1_	LG_2_	LG_3_	LG_4_
LA_1_	(0.75，0.21)	(0.16，0.47)	(0.57，0.13)	(0.54，0.25)
LA_2_	(0.52，0.34)	(0.23，0.35)	(0.46，0.22)	(0.49，0.34)
LA_3_	(0.69，0.27)	(0.54，0.26)	(0.32，0.35)	(0.38，0.41)
LA_4_	(0.83，0.12)	(0.24，0.63)	(0.65，0.29)	(0.74，0.23)
LA_5_	(0.48，0.51)	(0.32，0.58)	(0.58，0.18)	(0.63，0.34)

Step 2 Normalize the $IFDM={\left[IFDM_{ij}\right]}_{5\times 4}$ into $NIFDM={\left[NIFDM_{ij}\right]}_{5\times 4}$ (See Table 6).

**Table 6:** The $NIFDM={\left[NIFDM_{ij}\right]}_{5\times 4}$.

	LG_1_	LG_2_	LG_3_	LG_4_
LA_1_	(0.75，0.21)	(0.47，0.16)	(0.57，0.13)	(0.54，0.25)
LA_2_	(0.52，0.34)	(0.35，0.23)	(0.46，0.22)	(0.49，0.34)
LA_3_	(0.69，0.27)	(0.26，0.54)	(0.32，0.35)	(0.38，0.41)
LA_4_	(0.83，0.12)	(0.63，0.24)	(0.65，0.29)	(0.74，0.23)
LA_5_	(0.48，0.51)	(0.58，0.32)	(0.58，0.18)	(0.63，0.34)

Step 3 Obtain the IFNPIS (See Table 7).

**Table 7:** The IFNPIS.

	LG_1_	LG_2_	LG_3_	LG_4_
IFNPIS	(0.36，0.23)	(0.12，0.86)	(0.25，0.49)	(0.31，0.42)

Step 4 Obtain the IFNCSM information between $NIFDM_{ij}$ and $IFNPIS_{j}$ (See Table 8).

**Table 8:** The IFNCSM.

Alternatives	LG_1_	LG_2_	LG_3_	LG_4_
LA_1_	0.4213	0.5180	0.3280	0.5571
LA_2_	0.5802	0.3018	0.5945	0.2753
LA_3_	0.6200	0.1635	0.6099	0.3084
LA_4_	0.6048	0.5733	0.6150	0.5839
LA_5_	0.5123	0.3920	0.6150	0.6099

Step 5 Obtain the weight vectors (See Table 9):

**Table 9:** The weight vectors.

	LG_1_	LG_2_	LG_3_	LG_4_
weight	0.2035	0.3057	0.2475	0.2433

Step 6 Obtain the $IFNWAAV_{i}$ (Table 10).

**Table 10:** The $IFNWAAV_{i}$.

	LA_1_	LA_2_	LA_3_	LA_4_	LA_5_
IFNWAAV	0.4608	0.4244	0.4021	0.5926	0.5247

Step 7 Calculate the $IFNWGMV_{i}$ (Table 11).

**Table 11:** The $IFNWGMV_{i}$.

	LA_1_	LA_2_	LA_3_	LA_4_	LA_5_
IFNWGMV	0.4515	0.3987	0.3466	0.5924	0.5153

Step 8 Calculate the $IFNOAV_{i}\left(a\right),IFNOAV_{i}\left(b\right),IFNOAV_{i}\left(c\right)$ (See Table 12).

**Table 12:** Three designed strategies.

	$IFNOAV_{i}\left(a\right)$	$IFNOAV_{i}\left(b\right)$	$IFNOAV_{i}\left(c\right)$
LA_1_	0.1937	2.4486	0.7699
LA_2_	0.1748	2.2058	0.6946
LA_3_	0.1590	2.0000	0.6318
LA_4_	0.2516	3.1829	1.0000
LA_5_	0.2208	2.7916	0.8776

Step 9 Obtain the $IFNOAV_{i}$ (See Table 13).

**Table 13:** The $IFNOAV_{i}$.

	LA_1_	LA_2_	LA_3_	LA_4_	LA_5_
IFNOAV	1.8522	1.6696	1.5160	2.4069	2.1116

Step 10 In light with $IFNOAV_{i}\left(i=1,2,3,4,5\right)$, the order is $LA_{4}>LA_{5}>LA_{1}>LA_{2}>LA_{3}$ and the optimal high-tech enterprise is $LA_{4}$.

### 4.2 Comparative analysis and discussion

Then, the IFN-CSM-CoCoSo is **randomly** compared with IFWA [89] and IFWG [84], DGIFWBM [90], IF-COPRAS [91], IF-TODIM [92], IF-PROMETHEE [93] and IF-FUCOM-WASPAS [94]. The comparative results are built in Table 14.

**Table 14:** Order of different techniques.

	Order
IFWA technique [89]	$LA_{4}>LA_{5}>LA_{1}>LA_{2}>LA_{3}$
IFWG technique [84]	$LA_{4}>LA_{5}>LA_{2}>LA_{1}>LA_{3}$
DGIFWBM technique [90]	$LA_{4}>LA_{5}>LA_{1}>LA_{2}>LA_{3}$
IF-COPRAS technique [91]	$LA_{4}>LA_{5}>LA_{1}>LA_{2}>LA_{3}$
IF-TODIM technique [92]	$LA_{4}>LA_{5}>LA_{2}>LA_{1}>LA_{3}$
IF- PROMETHEE technique [93]	$LA_{4}>LA_{5}>LA_{1}>LA_{2}>LA_{3}$
IF-FUCOM-WASPAS technique [94]	$LA_{4}>LA_{5}>LA_{1}>LA_{2}>LA_{3}$

In light with WS coefficients [95,96], the WS coefficient between IFWA [89], IFWG [84], DGIFWBM [90], IF-COPRAS [91], IF-TODIM [92], IF-PROMETHEE [93], IF-FUCOM-WASPAS [94] and the IFN-CSM-CoCoSo technique is 1.0000, 0.7917, 1.0000, 1.0000, 0.7917, 1.0000, 1.0000. This verifies the IFN-CSM-CoCoSo technique is effective.

## 5 Conclusion

The performance evaluation of CSR management is the central link of CSR work, directly related to the improvement and optimization of responsibility performance. It should be said that in recent years, Chinese enterprises have accumulated certain experience in evaluating the performance of CSR management; However, overall, Chinese enterprises do not have much or profound understanding of this system. Therefore, it is very important and necessary to conduct an in-depth exploration of foreign social evaluation, domestic social evaluation, and corporate self-evaluation techniques for evaluating the performance of CSR management. The performance evaluation of CSR from the perspective of environmental accounting is MAGDM. Recently, the CoCoSo and CSM was utilized to conduct MAGDM. The IFSs are an efficient tool for conducting uncertain information during the performance evaluation of CSR from the perspective of environmental accounting. In this study, the IFN-CSM-CoCoSo is done for MAGDM with IFSs. Finally, numerical study for performance evaluation of CSR from the perspective of environmental accounting is validated the proposed technique.

## Ethics declaration statement

The authors state that this is their original work, and it is neither submitted nor under consideration in any other journal simultaneously.

## Data availability

The authors agree that the data used in this manuscript is available to everyone and anyone can use this data by just citing this article.

## Funding information

This study did not receive any funding in any form.

## CRediT authorship contribution statement

**Jing Nie:** Writing – original draft, Methodology.

## Declaration of competing interest

The authors declare that they have no known competing financial interests or personal relationships that could have appeared to influence the work reported in this paper.
